# Otitis

**Published:** 1898-12

**Authors:** J. M. Woodson

**Affiliations:** Temple, Texas


For Texas Medical Journal.

### Otitis.

BY J. M. WOODSON, M. D., TEMPLE, TEXAS.

Read before the Brazos Valley Medical Association, at its sixth semi-annual meeting at Rockdale, Texas, Nov. 15 and 16, 1898.

It is my motive, in this brief paper, to set forth some or the leading points that will enable general practitioners to distinguish acute and inflammatory processes of the external auditory canal, from inflammation in the tympanic cavity.

It would seem that circumscribed external otitis should present no difficulty of diagnosis, but in some instances it is by no means easy. At first the patient complains of a sense of discomfort or uneasiness about the head, and may not be able to localize the pain. The ear may be ignored, and a carious tooth accused of causing the trouble. Inspection of the ear at this period may reveal nothing; if, however, we supplement our inspection by testing the sensitiveness of the canal by means of a cotton-tipped applicator, usually some point will be found where slight pressure will cause the patient to wince. It is very important that the ear should be inspected by reflected light, and the sensitiveness of the organ tested by the cotton-tipped applicator, before the introduction of an ear speculum, as a slight swelling near the meatus would probably escape notice after introducing the instrument. When the speculum is introduced, the sensitiveness of the deeper parts should be tested as above indicated.

Circumscribed external otitis usually affects the movable part of the canal; however the osseous portion may become involved. A localized swelling in the osseous portion should be looked on with a great suspicion, and especially if the swelling is confined to the posterior-superior region. It is in this region that the mastoid is separated from the meatus by a very thin plate of bone, and an inflammation in the mastoid cells often causes an encroachment on the lumen of the canal at this locality. When this condition exists, an otoscopic examination gives the impression of a very narrow fundus, and the line of demarkation between the drum membrane and the posterior-superior wall is very poorly defined. This condition usually indicates a collection of pus in the mastoid antrum, and always indicates involvement of the deeper structures. On the other hand, a furuncular inflammation is usually near the orifice of the external meatus, and if the speculum can be passed beyond the localized swelling, an unobstructed view can be had of the depth of the canal.

External manipulation will reveal considerable tenderness upon pressure in front of the tragus if the anterior wall is affected; in fact, as the entire fibro cort canal is moved by pressure in this region, this test is of great importance in making differential diagnosis between inflammations affecting the canal, as distinguished from those of the deeper parts; that is, of otitis media and mastoiditis. If the oracle be moved vigorously in every direction, and pressure made on the superior, posterior, anterior and inferior walls, it will be impossible not to elicit tenderness if we have a case of external otitis The occurrence of œdema over the mastoid may lead to the erroneous supposition that the osseous structures have become involved. This mistake need never be made if care is taken to test the tenderness over the mastoid itself, without communicating motion to the auricle in applying the pressure. To do this, the fingers of the hand are rested on the head, while the thumb is pressed firmly on the œdematus area, taking care that the pressure is made just behind the insertion of the auricle and backward and inward, and not forward and inward. When the inflammation is limited to the canal, no tenderness will be evidenced by this pressure, although the thumb may sink into the œdematus mass, and leave its imprint when removed. As soon, however, as the pressure is directed forward, great pain is experienced. It is well to warn patients of the tendency of furuncles to appear in groups either collectively or successively, as one is relieved by spontaneous rupture or incision, another may appear, and in this way protract the suffering over several weeks. It is my practice to incise freely as soon as local tumefaction can be made out, and wash the canal frequently with a warm bichloride solution, 1 to 3000, and give constitutional treatment as the indications demand.

A saline cathartic, and frequent administration of camphorated tincture of opium, adds much to the comfort of the patient before the abscess is evacuated. Dry hot applications add somewhat to the relief of the pain, but the local application of drugs is usually disappointing. It is well to warn the patient against employing mechanical devices, such as ear spoons, tooth-picks, matches, and the like, for the purpose of relieving the itching of the canal, or to clear the canal of dried wax or other foreign substances, as these measures frequently abrade the epithelium and allow the canal to become affected and subject the patient to furuncular inflammation. While the great pain and loss of sleep may cause the patient profound systemic disturbance, we seldom find a rise of temperature above 101 degrees.

In acute catarrhal or supurative inflammation of the middle ear, we have, in addition to the intense pain, marked constitutional disturbances. The temperature frequently reaches 104-5, pulse very rapid, and in young children convulsions is not an infrequent occurrence. The difference of diagnosis between catarrhal and suppurative otitis media in the early stages is of the utmost importance, since the latter condition is always severe, being dangerous not only to the function of the organ, but to life itself. Particular attention should be given to the inspection of those parts lying above the short process of the malleus, whenever severe pain in the ear is complained of. It is the rule that in the early stages of suppurative otitis media, that portion of the membrana tympani alone lying above the short process of the malleus is the only part that presents the slightest deviation from normal appearance. In this locality the membrane is deeply congested. The hyperæmia does not extend below the posterior fold, and if a hasty examination is made it may escape observation. It is at this stage that prompt measures may abort the attack, hence the stress that is laid on physical characteristics. When seen later, marked engorgement is noticed, and the membrana flaccida is pushed outward and somewhat downward, and may overhang the short process. When well advanced, the hyperæmia is general, and may involve the entire drum membrane.

Now, in acute catarrhal inflammation, the entire drum membrane is hyperæmic. In the early stage a redness is most marked in the region of the manubrium, and shades off into normal color of the parts. A point of diagnostic value is that the entire membrane bulges as a whole, and a change in position not being limited to the membrane flaccida, whereas, in purulent inflammations involves the upper part of the cavity first, and causes a bulging of the membrane above the short process.

In case of catarrhal otitis the first indication is the relief of pain. Put the patient to bed, administer saline cathartic and sufficient opium to secure six or eight hours sleep, during which time from two to four ounces of blood should be taken from immediately in front of the tragus. In many instances this treatment will abort the attack. Dry heat is very grateful, and does not interfere with the measures employed to prevent the progress of disease. It is unwise to instill any oily solutions into the canal for the relief of pain, as they have no therapeutic value, and serve to obscure the parts when examination is made. Aqueous solutions of morphine, cocaine, atropine, or carbolic acid and glycerine, in proportion of 1 to 20, may be used in the canal without damage, and in many instances has a favorable influence over pain. After twelve or eighteen hours, if the above means have failed to abort the attack, and the pain continues, the membrana tympani should be freely incised. A free incision gives immediate and permanent relief. The canal should be rendered thoroughly aseptic before the incision is made. In the absence of positive indication for the point of incision, the knife should be inserted in the membrane below the posterior fold, and a curved incision made downward and forward to the interior pole. Great advantage is gained by inserting the knife into the internal wall, and thus depleting the engorged vessels in this region. The ear should be cleansed with a syringe from four to six times a day, using warm, sterile water each time. The patient should report to the physician once a day, at which time the ear is thoroughly cleansed by syringing and the use of air douches, or the ustachian catheter. If the physician can be seen daily, it is well to dust the canal with a dry antiseptic powder, but under no circumstances should the canal be filled with powder, or the use of powders be entrusted to the home attendant.

In suppurative otitis media, vigorous measures must be instituted at the beginning, if we hope to abort the attack. If, in the course of acute infectious disease, severe pain is complained of in the ear, and the characteristic congestion above mentioned is noticeable, immediate local depletion should be instituted. As much blood as the general condition of the patient will permit should be abstracted from the region in front of the tragus. The administration of an opiate to relieve pain is not advisable in these cases, since whatever measures are to be instituted for the relief of the local condition must be employed in the course of a few hours, and it is unwise to mask any advance of the disease by blunting the susceptibility of the patient to the intensity of pain. If depletion does not give immediate relief, the part should be thoroughly incised. This operation is intensely painful, but quickly performed, and the wisdom of administering a general anæsthetic depends upon the condition of the patient. The local application of cocaine scarcely renders the procedure less painful. The incision should lie above the short process of the malleus, and posterior to it. The knife is entered just behind the processus brevis, and carried upward and inward, parallel to the neck of the malleus, until it has pierced the cellular tissue within the tympanic vault, and impinges on the bony wall of the canal. The knife is then carried backward to the periphery of the membrane, the deep structures being incised throughout the entire extent of the incision. It is well, on reaching the periphery, to extend the incision on the posterior-superior wall for the distance of one-fourth of an inch, cutting through the soft parts of the bone. The free bleeding which follows this incision should be encouraged by irrigating with hot sterile water. It is not intended to liberate pus by the procedure, but to prevent its formation, hence the greatest antiseptic precaution should be observed, to prevent infection. These measures, when resorted to sufficiently early, may abort an attack of suppurative otitis media.

The wound made by this incision heals in a few days, and all pain referable to the ear disappears in four or five days. If the case is not seen until the tissues begin to bulge, the incision should begin over the area of the greatest bulging. Carry the knife into the tympanic vault, and divide the parts upward to the tympanic ring. The peripheral attachment should then be followed downward for a short distance, thus making a triangular flap, and thereby securing perfect drainage. The canal should be frequently irrigated with hot steril water, and the patient carefully watched in order to early recognize any extension of the disease to the mastoid cells. If tenderness on pressure over the mastoid, and sagging of the posterior-superior wall of the canal, should occur, complicating mastoiditis is to be suspected, and vigorous measures instituted to prevent the development of this complication. A saline should be given, the patient confined to bed. The aural ice bag or Leiter's coil applied over the mastoid process, and kept in position for 24 or 36 hours. If the posterior-superior incision has closed, it should be reopened. In the event these measures do not relieve the mastoid symptom, and the pain is severe and depression marked, we are not justified in further delay, but should enter the mastoid by the external route.

